# Recovery of FAM134A-mediated ER-phagy through BRD4 inhibition alleviates ethanol-induced neurodegeneration

**DOI:** 10.7150/ijbs.116673

**Published:** 2025-08-11

**Authors:** Jae Ryong Lim, Chang Woo Chae, Jee Hyeon Yoon, Ji Hyeon Cho, Ji Yong Park, Su Jong Han, Han Seung Chang, Su Yeol Kim, Ha Jin Kim, Young Hyun Jung, Ho Jae Han

**Affiliations:** 1 Department of Veterinary Physiology, College of Veterinary Medicine, Research Institute for Veterinary Science, and BK21 FOUR Future Veterinary Medicine Leading Education and Research Center, Seoul National University, Seoul, 08826, South Korea.; 2 Department of Physiology, and Brain Research Institute, College of Medicine, Chungnam National University, Daejeon 35015, South Korea.; 3 Department of Medical Science, College of Medicine, Chungnam National University, Daejeon 35015, South Korea.; 4 Department of Physiology, College of Medicine, Soonchunhyang University, Cheonan, 31151, Republic of Korea.

**Keywords:** BRD4, Endoplasmic reticulum stress, ER-phagy, Ethanol, FAM134A, Neurodegeneration

## Abstract

Endoplasmic reticulum (ER) stress is a major contributor to ethanol-induced neurodegeneration. ER-phagy, the selective elimination of specific ER domains, has emerged as a protective mechanism against ER stress. However, its regulation in ethanol-related neurological disorders remains unclear. Here, we investigated the effects and underlying mechanisms of ethanol on ER-phagy in neuronal cells and ethanol-fed mice. Our findings demonstrate that ethanol-induced ER stress is chronically sustained due to impaired ER-phagy. Among ER-phagy receptors, FAM134A expression was notably reduced by ethanol. Ethanol metabolism contributes to the downregulation of SIRT1 activity, leading to increased acetylation of histone H4 lysine 16 (H4K16ac) and enhanced recruitment of bromodomain-containing protein 4 (BRD4) to the FAM134A promoter. The BRD4/G9a complex-mediated increase in histone H3 lysine 9 dimethylation (H3K9me2) downregulates FAM134A expression by restricting the access of unfolded protein response (UPR)-associated transcription factor XBP1s. BRD4 inhibition or FAM134A overexpression restored ethanol-decreased ER-phagy, alleviating ER stress and preventing synaptic loss and neuronal cell death. In ethanol-fed mice, pharmacological inhibition of BRD4 restored hippocampal ER-phagy, resulting in improved cognitive function. In conclusion, recovering FAM134A-mediated ER-phagy through BRD4 inhibition may be a promising strategy to prevent ethanol-induced neurodegeneration.

## Introduction

Excessive alcohol consumption causes neurological problems, including synaptic degeneration and neuronal cell death 1-4. A key contributor to ethanol-induced neurological disorders is chronic endoplasmic reticulum (ER) stress, which disrupts cellular homeostasis 5-7. ER-phagy, also known as reticulophagy, is a selective form of autophagy that degrades specific portions of the ER 8. This tightly regulated process helps alleviate ER stress by removing unfolded/misfolded proteins within ER or by reducing the volume of the expanded ER 9, 10. During ER-phagy, receptors containing LC3-interacting regions (LIRs) recruit autophagy machinery to form autophagosomes, which subsequently fuse with lysosomes for degradation 11, 12. To date, several transmembrane ER-phagy receptors have been identified in mammals, such as FAM134A, FAM134B, FAM134C, RTN3L, CCPG1, SEC62, TEX264, and ATL3 13. Increasing evidence suggests that these receptors are crucial for maintaining ER homeostasis. A previous study reported that SEC62 facilitates the elimination of excessive proteins in order to maintain ER homeostasis 14. Another study showed that CCPG1 expression is upregulated in response to ER stress, triggering degradation of the ER membrane and associated proteins 15. These findings indicate that ER-phagy may play a key role in preventing ethanol-induced neurodegeneration by relieving ER stress. Nevertheless, the precise signaling pathways and molecular mechanisms underlying ethanol-induced ER-phagy remain incompletely understood.

Ethanol-induced epigenetic changes, such as histone modifications and DNA methylation, can activate or repress the expression of autophagy-related genes, thereby influencing neuronal survival 16-19. A recent study reported that acetate, a metabolite of ethanol, is directly incorporated into histones, leading to increased histone acetylation in the brain 20. This finding suggests that histone acetylation plays a key role in modulating ethanol-induced gene expression changes in neuronal cells. Histone modifications are interpreted by epigenetic reader proteins that regulate gene expression by recognizing specific epigenetic marks 21, 22. Among these, bromodomain-containing proteins selectively bind to acetylated lysine residues on histones to influence transcription 23. Bromodomain-containing protein 4 (BRD4), in particular, has traditionally been characterized as a transcriptional coactivator that recruits factors such as positive transcription elongation factor b (P-TEFb) and the Mediator complex. 24. However, recent studies have revealed that BRD4 plays a more versatile role in transcription control. Sakamaki et al. demonstrated that BRD4 acts as a repressor to suppress gene expression by binding to acetylated histone H4 lysine 16 sites (H4K16ac) on autophagy-related gene promoters 25. Furthermore, BRD4 has been shown to inhibit the expression of genes involved in mitophagy, aggrephagy, and pexophagy 25-27. Considering BRD4's role in repressing autophagy-related gene expression and the effects of ethanol metabolism in increasing histone acetylation, we hypothesize that BRD4 may inhibit ER-phagy under ethanol exposure. In this study, we investigated the mechanisms by which ethanol modulates neuronal ER-phagy in human induced-pluripotent stem cell-derived neurons (iPSC-neurons) and SH-SY5Y human neuroblastoma cells. We also examined the effect of recovering ER-phagy on cognitive function in ethanol-fed ICR mice.

## Materials and Methods

### Materials

Fetal bovine serum (FBS) was purchased from Hyclone (Logan, UT, USA) and antibiotics were purchased from Gibco (Grand Island, NY, USA). JQ1 (SML1524), N*-*acetyl cysteine (NAC; A7250), Sodium phenylbutyrate (PBA; SML0309), Diallyl disulfide (SMB00378), SRT1720 (567860), BIX01294 (B9311), and PSD95 antibody (MAB1596) were obtained from Sigma-Aldrich (St. Louis, USA). Small interfering RNAs (siRNAs) for *FAM134A*, *DDRGK1*, and non-targeting (NT) were purchased from Bioneer. Synaptophysin antibody (ab32127) was obtained from Abcam. The antibodies of CKAP4 (sc-393544), BRD4 (sc-518021), Calnexin (sc-23954), and β-Actin (sc-47778) were acquired from Santa Cruz. The antibodies of mCherry (NBP2-25157), IRE1 (NB100-2324), p-IRE1 (Ser724) (NB100-2323), and FAM134C (NBP1-60104) were purchased from Novus Biologicals. The antibodies of BRD2 (5848), BRD3 (94032), XBP1s (40435), CHOP (2895), Caspase-9 (9508), Cleaved Caspase-3 (9661), PSD95 (36233), LAMP1 (9091), BRD4 (13440), H3K9me2 (4658S), EIF2α (9722), p-EIF2α (Ser51) (9721), and FAM134B (83414) were bought from Cell Signaling Technology. The antibodies of PERK (A18196), p-PERK (Thr982) (AP0886), H4K16ac (A23091), KAT8/MYST1/MOF (A3390), G9a (A1247), ATL3 (A12196), RTN3 (A15129), and SEC62 (A18589) were purchased from ABclonal. The antibodies of CCPG1 (13861-1-AP) and TEX264 (25858-1-AP) were acquired from Proteintech. FAM134A antibody (MBS9235266) was acquired from Mybiosource.

### Cell culture

The iPSCs were obtained from the National Stem Cell Bank of Korea and Korean Cell Line Bank (KSCBi005-A). Induction of neural stem cells (NSCs) from iPSCs was carried out by culturing the cells on plates coated with human vitronectin (Thermo Fisher, Waltham, USA, A14700) in neural induction medium (Thermo Fisher, A1647801). Following NSC induction, the cells were replated onto dishes pre-coated with poly-L-ornithine (Sigma, Massachusetts, USA, P3655) and laminin (Thermo Fisher, 23017). NSCs were then maintained in neural differentiation medium composed of Neurobasal medium (Thermo Fisher, 21103) supplemented with 1% GlutaMax-1 (Thermo Fisher, 35050) and 2% B27 serum-free supplement (Thermo Fisher, 17504) for three weeks. To enhance neuronal differentiation, 5 mM dibutyryl-cAMP (Sigma, D0627) was added to the differentiation medium between days 7 and 10. Mouse hippocampal neuron cultures were performed according to a modified version of the previously described protocol 28. Hippocampal neurons from E18 mouse embryos were used in accordance with the guidelines and approval of the Institutional Animal Care and Use Committee at Seoul National University (SNU-231109-1-1). In brief, hippocampal neurons were grown at low density on poly-D-lysine-coated coverslips with cortical rings of neurons and glia or high density on poly-D-lysine-coated six-well plates in neurobasal medium supplemented with 2% B27 supplement and 0.25% GlutaMax-1. The SH-SY5Y human neuroblastoma cells were obtained from the Korean Cell Line Bank (Seoul, Korea). Since SH-SY5Y cells have neuronal characteristics, high stability, and reproducibility, they have been widely used to investigate the pathogenesis of neurodegenerative diseases. SH-SY5Y cells were cultured in high-glucose Dulbecco's Modified Eagle Medium (DMEM) containing 10% fetal bovine serum (FBS) and 1% antibiotics at 37 °C in a humidified incubator with 5% CO₂. Once the cells reached approximately 80% confluence, the culture medium was replaced with serum-free medium supplemented with 1% antibiotics and incubated for 24 h before drug treatment. SH-SY5Y cells were specifically used for plasmid-based experiments due to their high transfection efficiency and reproducibility compared to iPSC-neurons.

### Western blot analysis

Cells or tissues were harvested and lysed by incubating on ice for 30 min in RIPA lysis buffer (ATTO Corporation, Tokyo, Japan, WSE-7420) supplemented with protease and phosphatase inhibitors (Thermo Fisher, 78440). Cell lysates were centrifuged at 12,000 rpm for 20 minutes at 4 °C to remove insoluble debris. Protein concentrations were measured using a BCA assay kit (Thermo Fisher, 23227). Equal amounts of total protein were loaded onto 8-15% SDS-PAGE gels for separation and then transferred to PVDF membranes. Membranes were incubated in 5% skim milk for 40 min to block nonspecific binding (Gibco, 232100), followed by washing four times with TBST solution, each wash lasting 7 min. Membranes were incubated with primary antibodies overnight at 4 °C, followed by washing and subsequent incubation with HRP-conjugated secondary antibodies (1:10,000) for 2 h at room temperature. Western blot signals were visualized using chemiluminescence (BioRad, Hercules, CA, USA), and densitometric analysis was conducted using Image J software (Wayne Rasband, National Institutes of Health, Bethesda, MD, USA).

### Immunofluorescence analysis

Cells were fixed in 4% paraformaldehyde for 5 min, then permeabilized with 0.1% Triton X-100 for 5 min. To minimize nonspecific antibody binding, cells were incubated with 5% normal goat serum for 1 h at room temperature. Cells were incubated with primary antibodies for 24 h at 4 °C. After washing three times with PBS, cells were incubated with fluorescently labeled secondary antibodies (1:300 dilution) for 2 h at room temperature. Images were captured using either a super-resolution radial fluctuations (SRRF) imaging system (Andor Technology, Belfast, UK) or a confocal microscope system (LSM 710, Carl Zeiss, Oberkochen, Germany). Fluorescence intensities were quantified using Fiji software.

### Transfection of plasmid DNA

The pcDNA3.1-FAM134A-GFP or pcDNA3.1-GFP was transfected into SH-SY5Ys. Cells were incubated with a mixture of plasmid DNA, opti-MEM (Gibco, 31985062), and lipofectamine 2000 (Thermo Fisher Scientific, 11668019). After incubation for 12 h, lipofectamine reagent was removed and cells were incubated with fresh media. The plasmids pcDNA3.1-FAM134A-GFP and pcDNA3.1-GFP were manufactured by VectorBuilder and Koma Biotech, respectively. Transfection efficiency was confirmed by western blot in Supplementary Fig. 1A.

### EATR assay

EATR exploits the decreased stability of eGFP compared to mCherry in the acidic environment of lysosomes. Cells were transfected with 3 μg of Tet-On-mCherry-eGFP-RAMP4, and doxycycline was added at 4 µg/ml for 24 h to induce the expression of mCherry-eGFP-RAMP4. All EATR experiments used live cells without fixation, and the fluorescence was visualized with confocal microscopy. The Tet-On-mCherry-eGFP-RAMP4 (#109014) was acquired from Addgene.

### CCER assay

CCER assay utilizes the principle that free mCherry accumulates within lysosomes for long times before degradation due to the protease resistance of mCherry. The pcDNA3.1- RAMP4-mCherry vectors were transfected into SH-SY5Ys. To quantify ER-phagy, we performed western blotting and then measured the band intensities of mCherry-RAMP4 and free mCherry. For robust visualization of free mCherry, high amounts of protein (30 μg per well) were loaded. The pcDNA3.1-RAMP4-mCherry was purchased from Vectorbuilder.

### Small interfering RNA (siRNA) transfection

Cells were incubated with 40 nM of the specific siRNAs and transfection reagent TurboFect (Thermo Fisher, R0531) for 24 h without antibiotics. The culture medium was replaced with serum-free high-glucose DMEM. The siRNAs specific for *FAM134A*, *DDRGK1*, and nontargeting (NT) were purchased from Bioneer Corporation (Daejeon, Korea). siRNA efficiency was confirmed by western blot in Supplementary Fig. 1B.

### Chromatin immunoprecipitation (ChIP) assay

ChIP assay was conducted using EpiQuik™ Chromatin Immunoprecipitation Kit (Epigentek, p-2002) according to the manufacturer's instructions. Samples were incubated with antibody, the RNA polymerase, and the normal IgG for 90 min. Normal IgG and RNA polymerase were used as negative control and positive control, respectively. Sample DNA was extracted and amplified by quantitative real-time PCR using a designed primer. The sequences of FAM134A primer are as follows: forward primer, 5′-GGG ATA CTG GGA ATC TGG AA-3′ and reverse primer, 5′-CCC GTT TTC TTA TCC CAC A-3′. Amplified PCR products were separated by agarose gel electrophoresis using gels containing SafeView nucleic acid stain and visualized under UV light. Band intensities were quantified using ImageJ software, and results were expressed as fold changes relative to the control group. Statistical analyses were conducted using the quantified values to assess significance between experimental conditions.

### NAD^+^/NADH Assay

Intracellular NAD^+^ and NADH levels were measured by NAD^+^/NADH Assay Kit (Abcam, ab65348) following manufacturer's instructions. Briefly, cells were washed with cold PBS and lysed with NAD^+^/NADH extraction buffer. The sample was placed on ice for 20 min, followed by 10 min at room temperature. Total NAD (NADt) and NADH were measured according to the instructions, with absorbance read at 450 nm. NAD^+^/NADH ratio is calculated as: [NADt - NADH]/NADH.

### SIRT1 activity assay

SIRT1 activity was measured using the SIRT1 activity assay kit (Abcam, ab156065) according to the manufacturer's instructions. Briefly, cells were collected and lysed using a lysis buffer. The reactions were initiated by adding the cell lysates into a reaction mixture that included SIRT1 assay buffer, fluoro-substrate peptides, and NAD^+^. Fluorescence intensity was measured continuously for 1 h at 2 min intervals, with an excitation wavelength of 340 nm and an emission wavelength of 460 nm, using a microplate reader.

### Co-immunoprecipitation

Cells were lysed using Pierce™ IP Lysis Buffer (Thermo Fisher, 87788) containing protease inhibitors. Primary antibodies were immobilized on protein G magnetic beads (Sure Beads, Bio-Rad, CA, USA, 161-4021), and these antibody-bound beads were incubated with the cell lysates at 4 °C for 12 h. Subsequently, beads were washed three times using PBST and proteins were eluted with 20 mM glycine buffer (pH 2.0) for 5 min. Eluted proteins were neutralized with 1 M phosphate buffer and prepared with Laemmli sample buffer for further analysis.

### Reverse transcription-PCR and real-time PCR

Total RNA was isolated from cells using the RNA Extraction Kit (TaKaRa, Japan, 9767). Reverse transcription-PCR (RT-PCR) was performed with 1 μg of RNA using the Maxime RT-PCR premix kit (iNtRON Biotechnology, Sungnam, Korea, 25081). RT-PCR was conducted at 45 °C for 1 hour for cDNA synthesis, followed by enzyme inactivation at 95 °C for 5 min. The synthesized cDNA was amplified in a Rotor-Gene 6000 real-time thermal cycler (Corbett Research, Mortlake, Australia) using gene-specific primers and TB Green Premix Ex Taq (TaKaRa, RR420A). Real-time quantitative PCR cycling conditions were as follows: initial polymerase activation at 95 °C for 10 min, followed by 50 cycles consisting of denaturation at 94 °C for 15 sec, annealing at 54 °C for 30 sec, and extension at 72 °C for 30 sec. Primers were sourced from Bioneer Corporation, and their specificity was confirmed by analyzing PCR products and melting curves. Relative mRNA expression was calculated using the Delta-Delta Ct method and normalized to the *ACTB* gene expression.

### Measurement of intracellular ROS

Cells were washed once with phosphate-buffered saline (PBS) and incubated with 1 μM CM-H_2_DCFDA at 37 °C for 20 min in the dark. After staining, cells were treated with 0.05% trypsin for 3 min, followed by centrifugation at 1,500 g for 5 min. The cell pellet was then washed once with PBS and resuspended in fresh PBS. Fluorescence signals from DCFDA-stained cells were analyzed using a flow cytometer (CytoFlex; Beckman Coulter, USA).

### LysoTracker and DQ-BSA staining

Cells were washed once with phosphate-buffered saline (PBS) and incubated with 50 nM LysoTracker Deep Red (Thermo Fisher, L12492) for 30 min at 37 °C in dark and 10 μg/ml DQ Green BSA (Thermo Fisher, D12050) 20 min at 37 °C in dark, respectively. LysoTracker and DQ-BSA signals were analyzed by flow cytometry.

### TUNEL assay

*In Situ* Cell Death Detection Kit (Roche, 11684795910) was employed to assess neuronal apoptosis. Cells were fixed in 4% paraformaldehyde for 15 min, permeabilized with 0.1% Triton X-100 in PBS for 5 min, and then incubated with the TUNEL reaction mixture for 1 h at 37 °C in the dark. Samples were imaged by confocal microscopy. TUNEL fluorescence intensity was measured using Fiji software.

### Annexin V/PI apoptosis assay

Cell apoptosis was assessed using an Annexin V/PI apoptosis detection kit (BD Biosciences, BD556547). Harvested cells were resuspended in the kit-provided binding buffer and stained with Annexin V-FITC and propidium iodide (PI) for 20 min at room temperature in the dark. Stained cells were analyzed by flow cytometry, and data were processed using CytExpert 2.3 software. Both Annexin V and PI negative cells (Q3) were classified as viable. Cells negative for Annexin V but positive for PI (Q1) were considered necrotic, while double-positive cells (Q2) were identified as late apoptotic. Annexin V-positive but PI-negative cells (Q4) were defined as early apoptotic. To avoid confusion with late apoptotic and necrotic cells, only the Q4 region was defined as the apoptotic fraction.

### Experimental design of the animal study

All animal experiments were conducted in accordance with the National Institutes of Health Guide for the Care and Use of Laboratory Animals and permitted by the Institutional Animal Care and Use Committee of Seoul National University (SNU-231109-1-1). Eight-week-old male ICR mice were obtained from OrientBio (Seongnam, Korea) and acclimated under standard laboratory conditions, including 20-25 °C temperature, humidity below 60%, and a 12 h light/dark cycle. The experiments were structured to create groups of equal size through randomization and blind analysis. The estimation of group size was carried out following the guidelines set by the Institutional Animal Care and Use Committee of Seoul National University (SD: 8%, expected effect size: 15%, number of groups: 4; alpha: 0.05, power (1-β): 0.8). Mice were randomly divided into four groups of the same size using block randomization, including the control group, ethanol-fed mice group, ethanol-fed + JQ1 injected mice group, and JQ1-injected mice group. They were freely allowed access to chow and water. After one week of acclimation, the control group received normal saline solution by an oral route daily for 4 weeks. The ethanol-fed mice group orally consumed 2 g/kg of 20% ethanol daily for 4 weeks to induce neurodegeneration 29. The ethanol-fed + JQ1-injected mice group was administered 25 mg/kg of JQ1 intraperitoneally (i.p.) to inhibit BRD4 activity, followed by ethanol administration 30 min later, daily for 4 weeks. The JQ1-injected mice group were intraperitoneally supplied with 25 mg/kg of JQ1 daily for 4 weeks. Following behavioral assessments, mice were anesthetized via intraperitoneal injection of alfaxalone (40 mg/kg) and xylazine (10 mg/kg), and subsequently euthanized for brain tissue collection.

### Open field test

The open field test is commonly used to evaluate anxiety levels in rodents, as they tend to stay near the edges of the open field when anxious. To minimize stress prior to testing, the animals were acclimated to the testing chamber for 2 h. Mice were placed in rectangular plastic boxes (H30 × L30 × W30 cm), and their movements were recorded for 10 min. The time spent in the center and periphery of the open field was analyzed using the Smart 3.0 video tracking system.

### Y-maze spontaneous alternation test

The Y maze test assesses spatial memory and learning in mice, which naturally prefer exploring a new arm of the maze rather than revisiting one they've already entered. To minimize stress, the mice were acclimated to the testing environment for 2 h before the experiment. Each mouse was randomly assigned to one of the Y maze's arms. Mice were allowed to freely explore the Y-maze for 8 min. Only if all four limbs were in the arm was entry deemed complete. We divide the number of triads by the maximum alternation (total entries-2) of 100 to get the percentage alternation. A higher alternation percentage indicates better spatial memory in the mice.

### Novel object recognition test (NOR)

The Novel object recognition (NOR) test relies on the natural tendency of mice to explore new objects more than familiar ones. Mice were first habituated to an open-field box for 5 min. After 24 h, they were allowed to freely explore the same open field with two identical objects for 10 min. After 4 h, one of the objects was replaced with a new one, and the mice were given another 10 min to explore the area. The discrimination index is one of the most commonly used parameters for evaluating cognitive function. DI represents the following: [(novel object exploration time - familiar object exploration time)/total exploration time]. An increase in the discrimination index indicates enhanced cognitive function. Novelty preference was assessed by calculating the percentage of time spent exploring the novel object relative to the total exploration time.

### Immunohistochemistry

Following deep anesthesia, mice underwent transcardial perfusion with PBS, followed by fixation with 4% PFA in 0.1 M phosphate buffer (pH 7.4). Brains were carefully extracted and post-fixed in 4% PFA for 2 h, then cryoprotected in 30% sucrose solution for 24 h at 4 °C. Coronal brain sections (40 μm thick) containing the hippocampus were obtained using a cryostat. Tissue sections were incubated in 5% NGS with 1% Triton X-100 for 1 h at room temperature for blocking, followed by overnight incubation with primary antibodies at 4 °C. Following washes, tissue sections were treated with suitable secondary antibodies for 2 h at room temperature. Fluorescent signals were visualized using a confocal microscope system (Carl Zeiss, LSM710). Given the specific involvement of the CA3 region in memory and neurodegenerative processes 30, this subregion was selected for analysis. Image analysis was performed using Fiji software.

### Statistical analysis

All quantitative data were expressed as mean ± standard deviation (SD). Statistical analysis was conducted using GraphPad Prism 6 software (GraphPad, CA, USA). Comparisons between two experimental groups were made using a two-tailed Student's t-test. One-way ANOVA followed by Tukey's post hoc test was used for multiple group comparisons. In cases where the sample size was less than 5, non-parametric test such as the Mann-Whitney U test was used instead. In such cases, for datasets with more than two groups, the Mann-Whitney U test was applied selectively for pairwise comparisons of interest. A p-value of < 0.05 was regarded as statistically significant.

## Results

### Effect of ethanol-induced ER stress on synaptic loss and neuronal cell death

**Figure 1:**
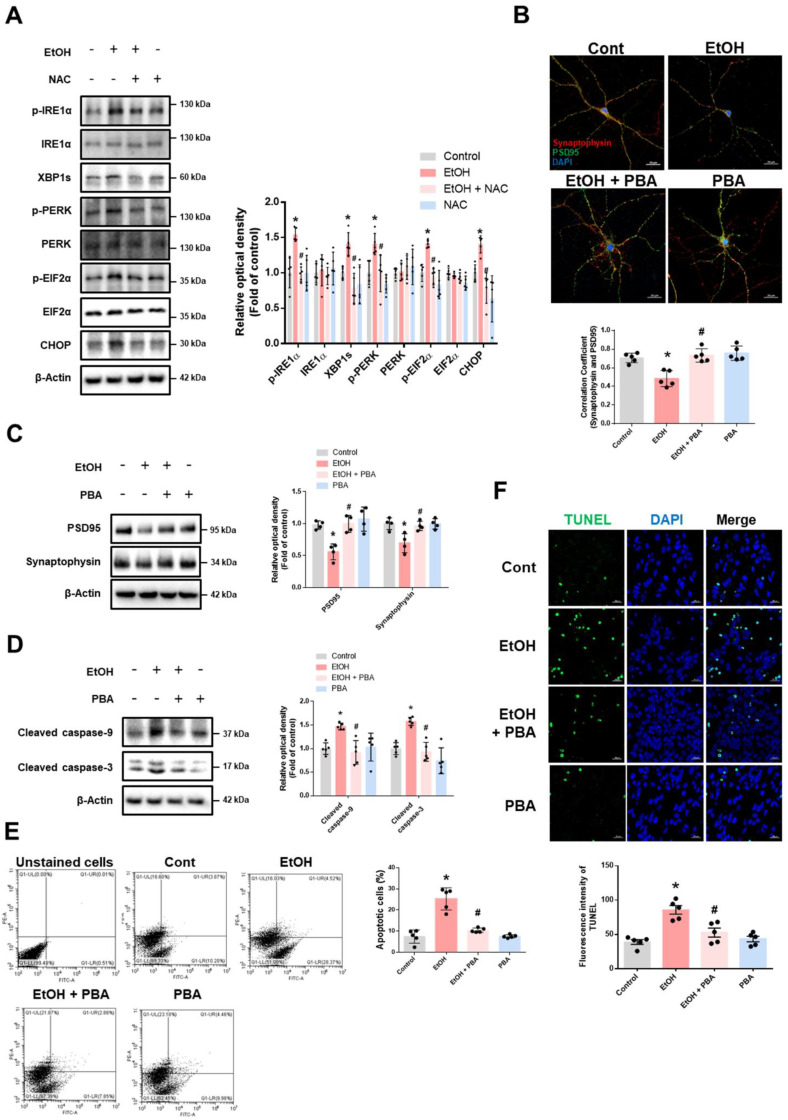
** Ethanol-induced ER stress promotes synaptic dysfunction and neuronal apoptosis. (A)** iPSC-neurons were pretreated with NAC (1 mM) for 30 min prior to EtOH (100 mM) for 24 h. ER stress marker proteins were analyzed by western blotting. β-Actin was used as a loading control. *n* = 5. **(B)** Hippocampal neurons were pretreated with PBA (5 mM) for 30 min prior to EtOH treatment (100 mM) for 48 h. Hippocampal neurons were immunostained with PSD95 (green), Synaptophysin (red), and DAPI (blue). Pearson's correlation coefficient was quantified for detecting synaptic density. Scale bars, 20 μm. *n* = 5. **(C-F)** iPSC-neurons were pretreated with PBA (5 mM) for 30 min prior to EtOH treatment for 48 h. **(C)** The protein levels of PSD95 and Synaptophysin were detected by western blotting. *n* = 4. **(D)** Expression of cleaved caspase-9 and cleaved caspase-3 were detected by western blotting. *n* = 5. **(E)** The annexin V/PI staining was used to measure apoptotic cells. *n* = 5. **(F)** Neuronal apoptosis was determined via TUNEL assay**.** The level of green fluorescence reflects the amount of neuronal cell death. Scale bars, 20 μm. *n* = 5. All data are representative. Quantitative data are presented as a mean ± SD. * indicates *p* < 0.05 versus control, # indicates *p* < 0.05 versus EtOH.

We first observed that ethanol increased the expression of ER stress marker proteins (Supplementary Fig. 2). Inhibition of enzyme involved in ethanol metabolism led to reduced ROS production (Supplementary Fig. 3). To assess the contribution of ROS on ethanol-induced ER stress, we examined whether ROS scavenging could reduce expression of ER stress marker proteins. Pretreatment with NAC effectively suppressed ethanol-induced ER stress (Fig. 1A), suggesting that ROS produced by ethanol metabolism is a primary driver of ER stress. To explore the role of ER stress in ethanol-induced synaptic dysfunction, we used the pharmacological ER stress inhibitor PBA. Ethanol treatment reduced synaptic density and the expression of synaptic marker proteins, both of which were restored by PBA pretreatment (Fig. 1B-C). Additionally, PBA mitigated ethanol-induced neuronal apoptosis (Fig. 1D-F). Overall, these findings indicate that ER stress triggered by ethanol contributes to synaptic dysfunction and neuronal cell death.

### Ethanol suppresses neuronal ER-phagy

**Figure 2:**
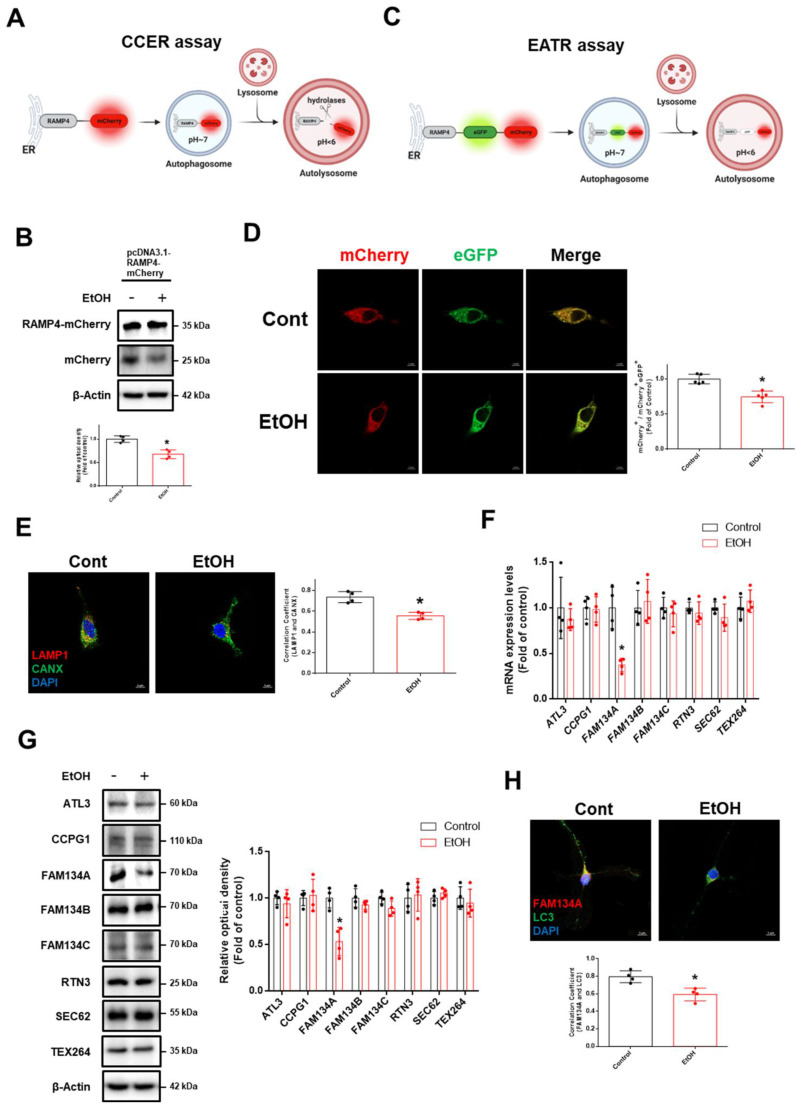
** Ethanol inhibits ER-phagy in neuronal cells. (A)** Schematic illustration of the mCherry-Cleaved ER-phagy Reporter (CCER) assay. When ER-phagy occurs, lysosomal cleavage of RAMP4-mCherry results in the formation of free mCherry product. **(B)** SH-SY5Ys were transfected with the pcDNA3.1-RAMP4-mCherry vector to establish the CCER system. The protein expression of mCherry and mCherry-RAMP4 were detected by western blotting.* n* = 4. **(C)** Schematic of the ER Autophagy Tandem Reporter (EATR) assay. When ER-phagy occurs, eGFP is quenched in autolysosome-induced low pH situations. **(D)** SH-SY5Ys were transfected with the Tet-On-mCherry-eGFP-RAMP4 vector to establish the EATR system and visualized after EtOH exposure (50 mM) for 24 h. Scale bars, 5 μm. *n* = 5. **(E)** Hippocampal neurons were exposed to EtOH for 24 h. Immunofluorescence staining of LAMP1 (lysosomal marker), CANX (ER marker)*,* and DAPI was visualized. Scale bars, 5 μm. *n* = 4. **(F)** iPSC-neurons were exposed to EtOH for 12 h. The mRNA expression levels of ER-phagy receptors were analyzed by using real time PCR. Data were normalized by the *ACTB* mRNA expression level. *n* = 4.** (G)** iPSC-neurons were treated with EtOH for 24 h. The protein levels of ER-phagy receptors were detected by western blotting. *n* = 4.** (H)** Hippocampal neurons were treated with EtOH for 24 h. Immunofluorescence staining of FAM134A, LC3, and DAPI was visualized. Scale bars, 5 μm. *n* = 4. All data are representative. Quantitative data are presented as a mean ± SD. * indicates *p* < 0.05 versus control.

We hypothesized that ethanol-induced ER stress was due to impaired ER-phagy. To quantitatively measure ER-phagy, we employed the recently developed mCherry-Cleaved ER-phagy Reporter (CCER) and ER Autophagy Tandem Reporter (EATR). The CCER system utilizes the principle that autophagosomes containing RAMP4-mCherry fuse with lysosomes, leading to RAMP4-mCherry cleavage in an acidic environment to generate free mCherry (Fig. 2A). We observed that ethanol induces a significant reduction in free mCherry expression (Fig. 2B). In the EATR system, cytosolic ER fluoresced in both the mCherry and eGFP, whereas ER in autolysosomes retained only mCherry fluorescence (Fig. 2C). Ethanol increased the ratio of green fluorescence compared to the control, indicating impaired ER-phagy (Fig. 2D). We also performed endogenous staining with lysosomal-associated membrane protein 1 (LAMP1) and calnexin (CANX) to confirm the effect of ethanol on the ER-phagy process. Ethanol reduced the interaction between LAMP1 and CANX (Fig. 2E). Further, we investigated which ER-phagy receptor was most affected by ethanol treatment. Ethanol significantly downregulated FAM134A mRNA and protein expression in neuronal cells (Fig. 2F-G). Additionally, ethanol reduced the interaction between FAM134A and microtubule-associated protein 1 light chain 3 (LC3), an autophagosome marker (Fig. 2H). Collectively, these results suggest that ethanol suppresses neuronal ER-phagy by downregulating FAM134A.

### BRD4 downregulates FAM134A expression through G9a-mediated H3K9me2

Given that ethanol metabolism increases histone acetylation and that bromodomain and extra-terminal (BET) family proteins recognize acetylated histones to regulate gene expression, we examined whether any of the BRD proteins are involved in modulating FAM134A expression in ethanol-exposed neuronal cells. We compared the binding of BRD2, BRD3, and BRD4 to the FAM134A promoter and found that ethanol markedly increased BRD4 binding, while BRD2 and BRD3 showed minimal association under both control and ethanol-treated conditions (Supplementary Fig. 4).

**Figure 3:**
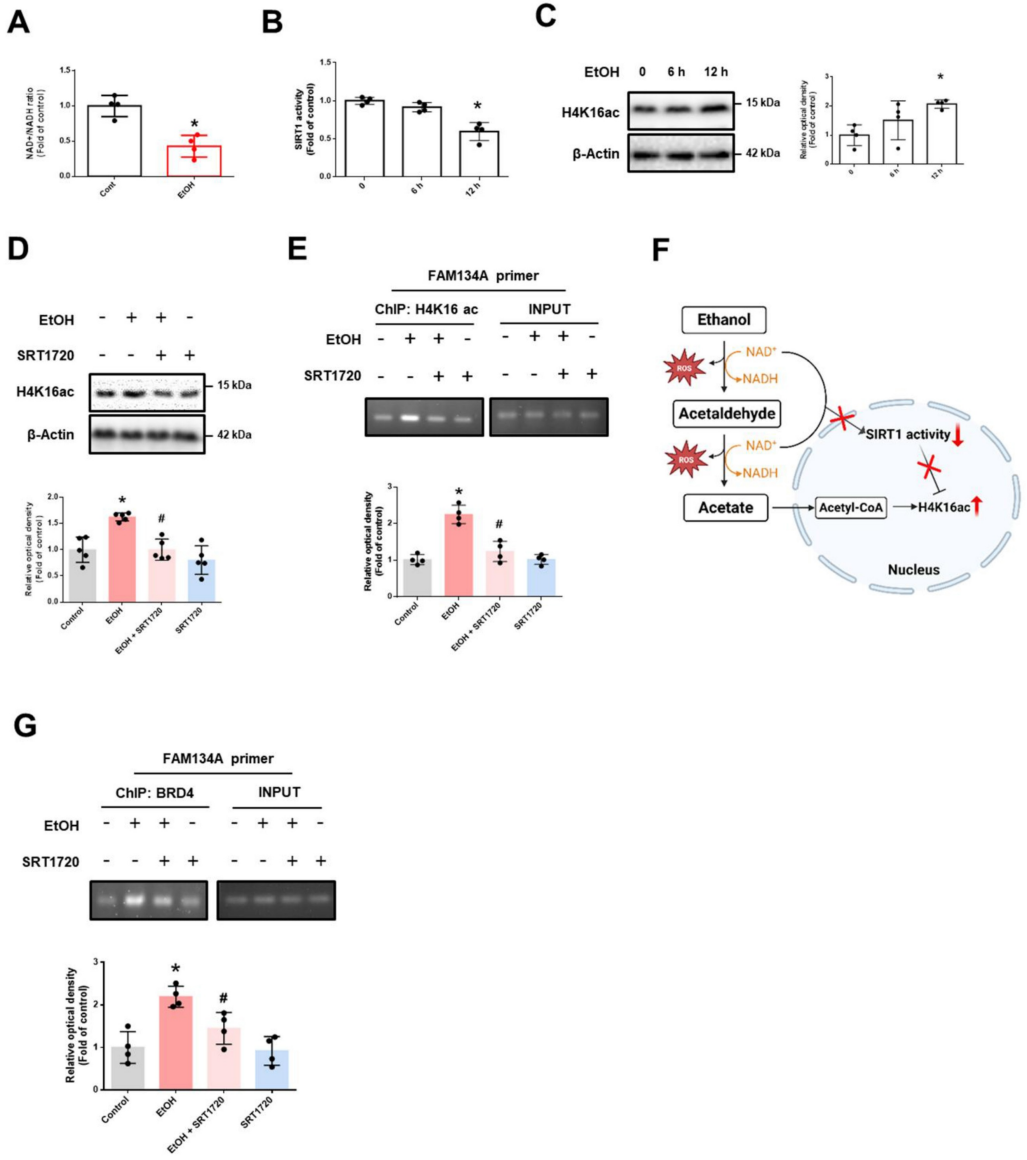
** Ethanol-induced downregulation of SIRT1 activity promotes BRD4 association with the FAM134A gene promoter. (A)** iPSC-neurons were exposed to EtOH for 12 h. NAD^+^/NADH ratio was determined using the NAD^+^/NADH assay kit. *n* = 4.** (B-C)** iPSC-neurons were treated with EtOH for 0, 6, and 12 h. **(B)** SIRT1 activity was measured with SIRT1 activity assay kit. *n* = 4.** (C)** Western blotting was conducted to determine the levels of H4K16ac. *n* = 4.** (D-G)** iPSC-neurons were pretreated with SRT1720 (1 μM) for 30 min prior to treatment of EtOH for 12 h.** (D)** H4K16ac levels were detected by western blotting. *n* = 5.** (E, G)** DNA was immunoprecipitated with H4K16ac or BRD4, respectively. The immunoprecipitation and input samples were amplified with primers of GAPDH and FAM134A**.** Data were analyzed by conventional PCR. *n* = 4. **(F)** Scheme illustrating the role of ethanol metabolism in the increase of H4K16ac levels. All data are representative. Quantitative data are presented as a mean ± SD. * indicates *p* < 0.05 versus control, # indicates *p* < 0.05 versus EtOH.

This finding prompted us to explore the mechanism by which BRD4 regulates FAM134A expression under ethanol exposure. The acetylation level of H4K16 is primarily controlled by the histone acetyltransferase hMOF and the nicotinamide adenine dinucleotide (NAD^+^)-dependent protein deacetylases SIRT1 31. Our results showed that ethanol decreased the NAD^+^/NADH ratio and the SIRT1 activity (Fig. 3A-B), but not protein expression of hMOF (Supplementary Fig. 5). These findings suggest that ethanol-induced increase in H4K16ac may be SIRT1-dependent. Additionally, ethanol increased H4K16ac levels in a time-dependent manner, which was inhibited by SRT1720 pretreatment (SIRT1-specific activator) (Fig. 3C-D). Additionally, SRT1720 reversed the ethanol-induced accumulation of H4K16ac on the FAM134A promoter (Fig. 3E). Given that acetate, a byproduct of ethanol metabolism, is a source of histone acetylation, we suggest that increased acetate along with decreased SIRT1 activity may contribute to H4K16 acetylation (Fig. 3F). Furthermore, ethanol-induced BRD4 binding to the FAM134A promoter was blocked by SRT1720 pretreatment (Fig. 3G). Collectively, ethanol-induced reduction in SIRT1 activity elevated H4K16ac on the FAM134A promoter, which in turn increases BRD4 binding.

**Figure 4:**
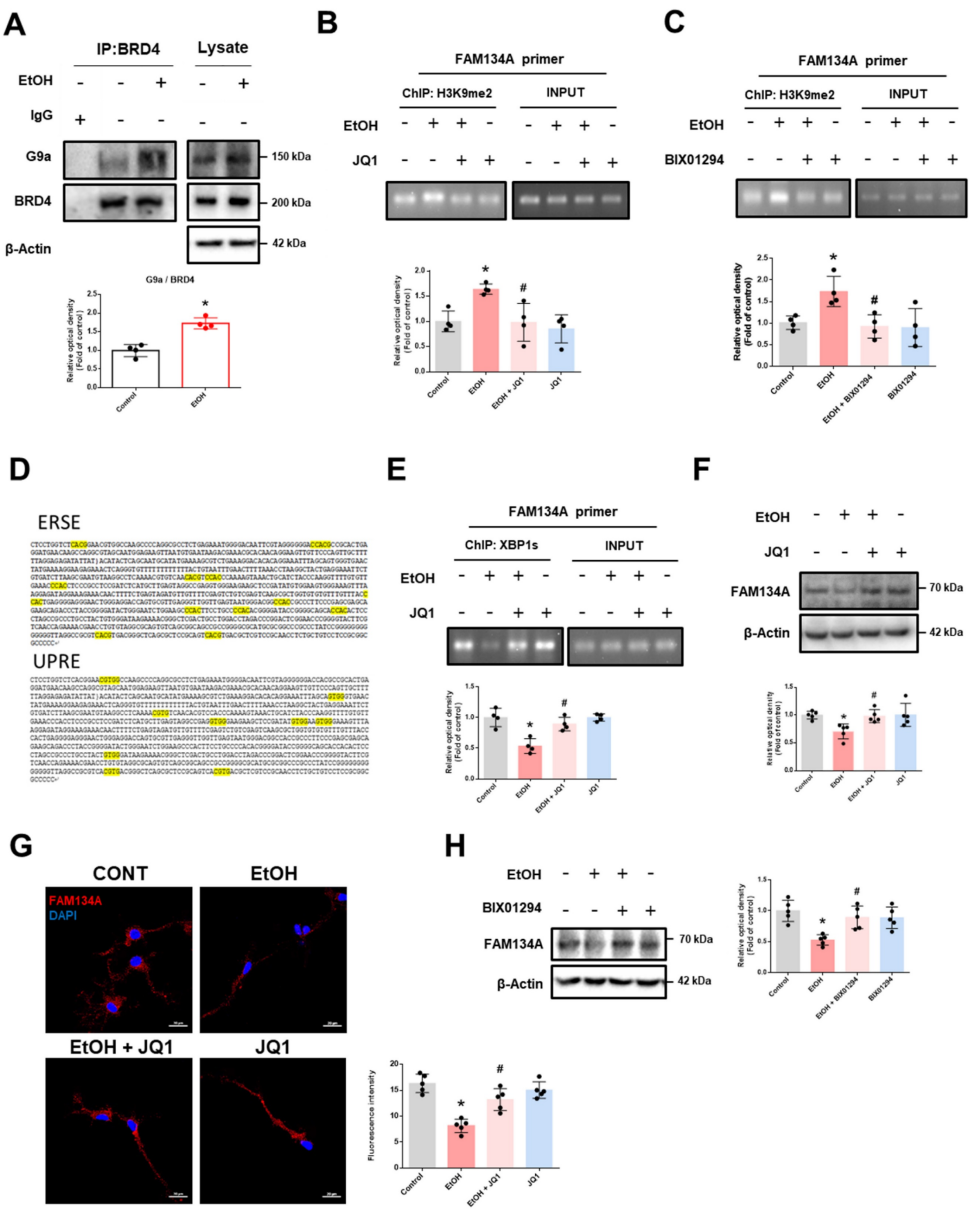
** BRD4 regulates FAM134A gene expression through G9a-mediated H3K9me2. (A)** iPSC-neurons were incubated with EtOH for 12 h. BRD4 was co-immunoprecipitated with anti-G9a antibody (the left panel). Total protein expression of G9a and BRD4 is shown in right panel. *n* = 4. **(B-C)** DNA was immunoprecipitated with H3K9me2. The immunoprecipitation and input samples were amplified with primers of GAPDH and FAM134A.** (B)** iPSC-neurons were pretreated with JQ1 (25 nM) for 30 min prior to treatment of EtOH for 12 h. *n* = 4. **(C)** iPSC-neurons were pretreated with BIX01294 (1 μM) for 30 min prior to treatment of EtOH for 12 h. *n* = 4.** (D)** 1000 base pairs upstream of the first codon of the FAM134A was described and the putative XBP1s binding sequences were emphasized with yellow labeling. **(E)** iPSC-neurons were pretreated with JQ1 (25 nM) for 30 min prior to treatment of EtOH for 12 h. DNA was immunoprecipitated with XBP1s. The immunoprecipitation and input samples were amplified with primers of GAPDH and FAM134A. *n* = 4. **(F-G)** iPSC-neurons were pretreated with JQ1 (25 nM) for 30 min prior to treatment of EtOH for 24 h. **(F)** The protein levels of FAM134A were detected by western blotting. *n* = 5.** (G)** Immunofluorescence staining of FAM134A was visualized. Scale bars, 20 μm. *n* = 5. **(H)** iPSC-neurons were pretreated with BIX01294 (1 μM) for 30 min prior to treatment of EtOH for 24 h. FAM134A expression was analyzed by western blotting. *n* = 5. All data are representative. Quantitative data are presented as a mean ± SD. * indicates *p* < 0.05 versus control, # indicates *p* < 0.05 versus EtOH.

We also explored how BRD4 binding to the FAM134A promoter contributes to gene repression. Recent studies have shown that BRD4 regulates gene expression by interacting with proteins that regulate histone methylation, such as G9a, enhancer of zeste homolog 2 (EZH2), and lysine-specific demethylase 1 (LSD1) 32, 33. We first confirmed that ethanol significantly upregulated G9a mRNA and protein levels in neuronal cells. (Supplementary Fig. 6A-B). Additionally, ethanol treatment enhanced the interaction between BRD4 and G9a (Fig. 4A). Inhibiting BRD4 and G9a significantly reduced ethanol-induced enrichment of H3K9me2 levels at the FAM134A promoter (Fig. 4B-C). Given recent evidence linking the unfolded protein response (UPR) with ER-phagy receptor expression 9, 34, we further identified binding regions for UPR-related transcription factors on the FAM134A promoter. We found several binding motifs of spliced X-box binding protein 1 (XBP1s), known as the ER stress response element (ERSE) including the CCACG sequence and the unfolded protein response element (UPRE) including the CGTGG sequence, in the FAM134A promoter region, 1000 bp upstream of the transcription start site (Fig. 4D). We also identified the activating transcription factor 4 (ATF4) binding motif, known as the cAMP Response Element (CRE), containing the TGACGTCA sequence, and the ATF6 binding motif containing the TGACGTG sequence (Supplementary Fig. 7A-B). Since the FAM134A promoter harbors more potential XBP1s binding sites than those for other UPR-related transcription factors, we hypothesized that XBP1s is a key regulator of FAM134A. ChIP assay results revealed that ethanol reduced XBP1s binding to the FAM134A promoter, which was restored by pretreatment with the BRD4 inhibitor JQ1 (Fig. 4E). These findings suggest that ethanol-induced BRD4-G9a complex-mediated H3K9me2 hinders XBP1s access to the FAM134A promoter. JQ1 pretreatment restored the ethanol-induced decrease in FAM134A mRNA expression (Supplementary Fig. 8). Furthermore, ethanol-induced downregulation of FAM134A expression was reversed by pretreatment with either JQ1 or BIX01294 (Fig. 4F-H). In summary, our results demonstrate that ethanol-induced SIRT1 activity reduction enhances the binding of H4K16ac and BRD4 on the FAM134A promoter, and subsequently suppresses FAM134A gene expression by blocking XBP1s access via the BRD4-G9a complex-mediated H3K9me2.

### Recovery of FAM134A-mediated ER-phagy prevents ethanol-induced synaptic dysfunction and neuronal apoptosis

**Figure 5:**
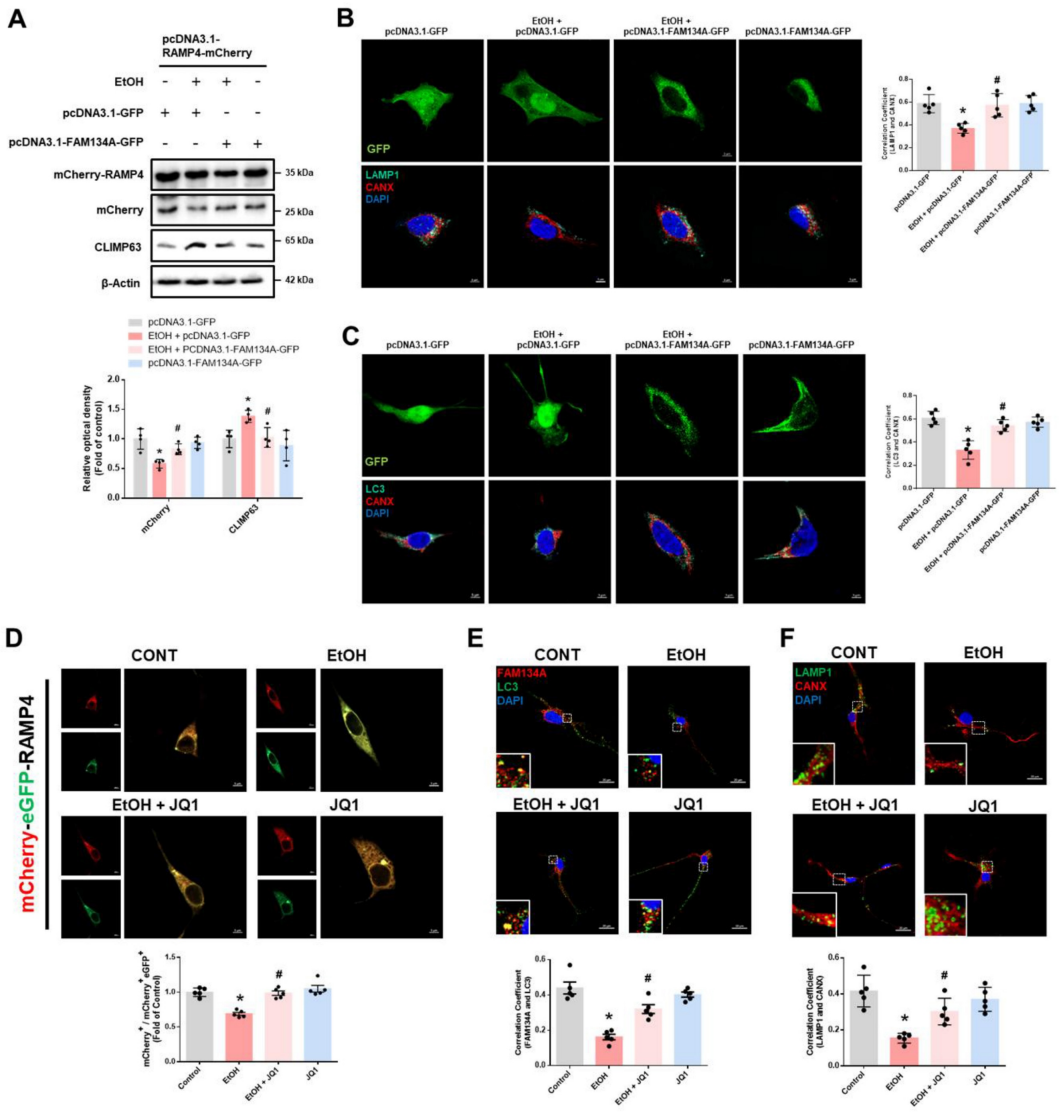
** Recovery of FAM134A alleviates ethanol-inhibited neuronal ER-phagy. (A)** After establishing the CCER system using pcDNA3.1-RAMP4-mCherry, SH-SY5Ys were additionally transfected with pcDNA3.1-FAM134A-GFP to overexpress FAM134A before exposure to EtOH for 24 h. The protein expression of mCherry-RAMP4, mCherry, and CLIMP63 (ER sheet marker) was detected by western blot. *n* = 4. **(B-C)** SH-SY5Ys were transfected with control vector or pcDNA3.1-FAM134A-GFP vector before exposure to EtOH for 24 h. **(B)** Immunofluorescence staining of LAMP1 and CANX was visualized. Scale bars, 5 μm, *n* = 5.** (C)** Immunofluorescence staining of LC3 and CANX was visualized. Scale bars, 5 μm, *n* = 5. **(D)** SH-SY5Ys were transfected with Tet-On-mCherry-eGFP-RAMP4 vector to establish the EATR system before EtOH exposure for 24 h. ER acidification was measured by confocal images. Scale bars, 5 μm, *n* = 5. **(E-F)** iPSC-neurons were pretreated with JQ1 (25 nM) for 30 min prior to treatment of EtOH for 24 h. **(E)** Immunofluorescence staining of FAM134A and LC3 was visualized. Scale bars, 20 μm, n = 5. **(F)** Immunofluorescence staining of LAMP1 and CANX was visualized. Scale bars, 20 μm, n = 5. All data are representative. Quantitative data are presented as a mean ± SD. * indicates *p* < 0.05 versus control, # indicates *p* < 0.05 versus EtOH.

**Figure 6:**
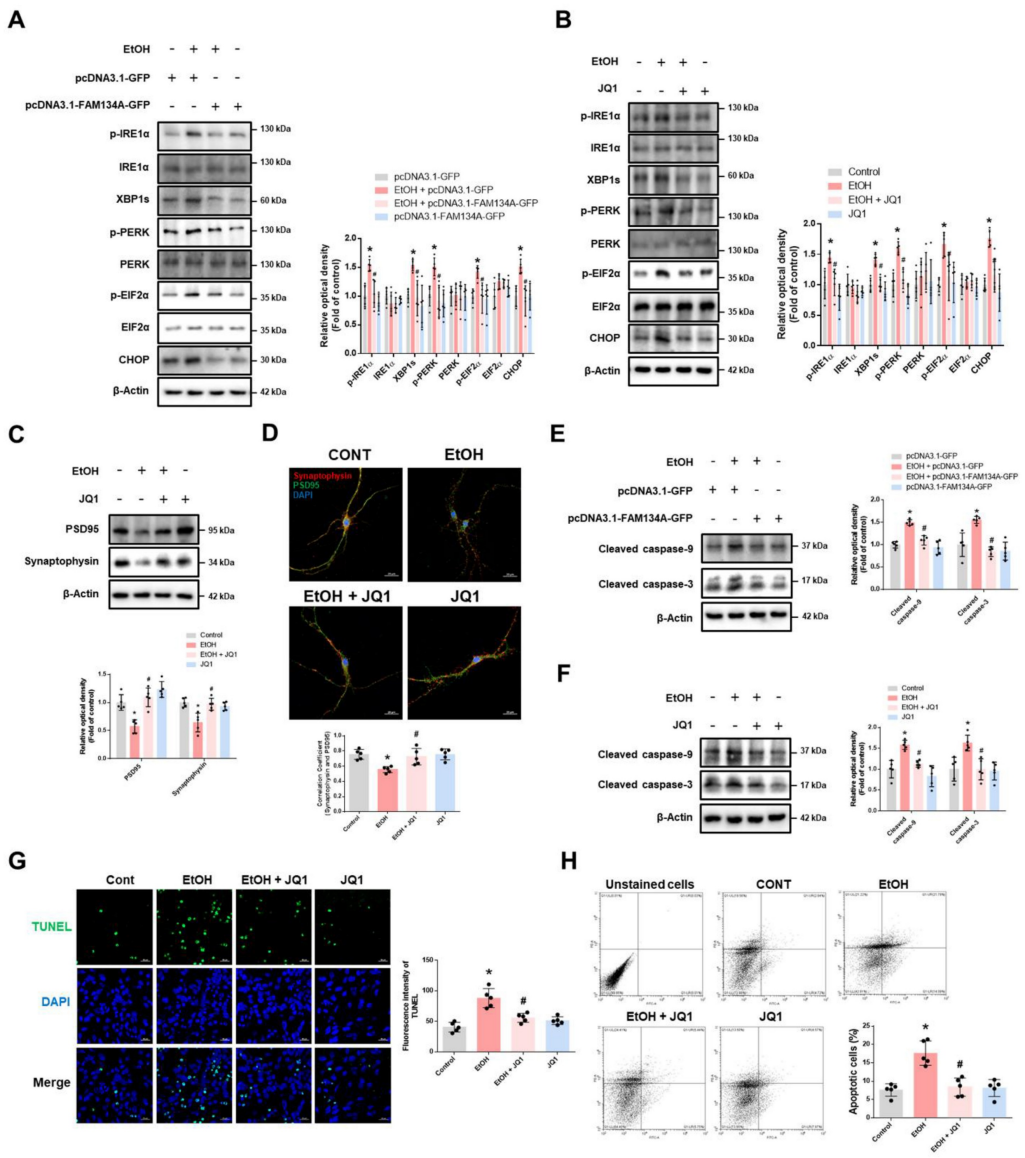
** Recovery of FAM134A suppresses ethanol-induced synaptic dysfunction and neuronal cell death. (A)** SH-SY5Ys were transfected with pcDNA3.1-GFP or pcDNA3.1-FAM134A-GFP before exposure to EtOH for 24 h. ER stress marker proteins were detected by western blotting. *n* = 5. **(B)** iPSC-neurons were pretreated with JQ1 (25 nM) for 30 min prior to treatment of EtOH for 24 h. Protein levels of ER stress markers were determined by western blotting.* n* = 5.** (C-D)** iPSC-neurons and hippocampal neurons were pretreated with JQ1 (25 nM) for 30 min prior to treatment of EtOH for 48 h, respectively.** (C)** Protein expression of PSD95 and Synaptophysin was analyzed by western blotting. *n* = 5. **(D)** Immunofluorescence staining of PSD95 and Synaptophysin was visualized with confocal microscope system. Scale bars, 20 μm. *n* = 5.** (E)** SH-SY5Ys were transfected with pcDNA3.1-GFP or pcDNA3.1-FAM134A-GFP before exposure to EtOH for 48 h. Western blot was performed to analyze the protein expression of cleaved caspase-9 and cleaved caspase-3. *n* = 5.** (F-H)** iPSC-neurons were pretreated with JQ1 (25 nM) for 30 min prior to treatment of EtOH for 48 h. **(F)** The protein levels of cleaved caspase-9 and cleaved caspase-3 were determined by western blotting. *n* = 5. **(G)** Neuronal apoptosis was assessed using a TUNEL assay. Scale bars, 20 μm. *n* = 5.** (H)** Apoptotic cells were measured by annexin V/PI analysis assay. *n* = 5. All data are representative. Quantitative data are presented as a mean ± SD. * indicates *p* < 0.05 versus control, # indicates *p* < 0.05 versus EtOH.

We next investigated whether restoring FAM134A expression, either through FAM134A overexpression or BRD4 inhibition, could reverse the ethanol-induced suppression of ER-phagy. FAM134A upregulation effectively rescued ER-phagy impaired by ethanol exposure (Fig. 5A-C). Additionally, BRD4 inhibition reversed the ethanol-induced ER-phagy impairment (Fig. 5D-F). In the CCER assay, JQ1 pretreatment increased free mCherry levels, which were reduced by ethanol, and this effect was suppressed by FAM134A silencing (Supplementary Fig. 9). These findings indicate that FAM134A plays a key role in restoring ER-phagy in ethanol-exposed neuronal cells. Additionally, BRD4 inhibition restored the expression of LAMP1, as well as the fluorescence intensity of LysoTracker and DQ-BSA, all of which were reduced by ethanol (Supplementary Fig. 10A-C). These results suggest that BRD4 inhibition not only restores FAM134A expression but also promotes lysosomal biogenesis, contributing to the recovery of ER-phagy function. Given recent findings that activation of ER-phagy receptors is critical for ER-phagy initiation, we further examined the regulatory mechanism of FAM134A activation. A previous study reported that ER-localized DDRGK1 regulates ER-phagy activation of the ER sheet through UFMylation 35. Because FAM134A is an ER-phagy receptor located on the ER sheet, we hypothesized that its activity may similarly depend on UFMylation. Indeed, although FAM134A overexpression restored ER-phagy reduced by ethanol, this effect was abolished when DDRGK1 was silenced (Supplementary Fig. 11). This suggests that FAM134A-mediated ER-phagy may be regulated by UFMylation. We also evaluated the protective role of FAM134A against ethanol-induced ER stress, synaptic dysfunction, and neuronal cell death. Both FAM134A overexpression and BRD4 inhibition decreased ethanol-induced ER stress (Fig. 6A-B). Moreover, ethanol-induced reductions in synaptic marker levels and synaptic density were prevented by JQ1 pretreatment (Fig. 6C-D). Lastly, FAM134A overexpression or JQ1 pretreatment prevented ethanol-induced neuronal apoptosis (Fig. 6E-H). Overall, these findings demonstrate that restoring FAM134A-mediated ER-phagy may alleviate ethanol-induced ER stress, preventing synaptic dysfunction and apoptosis in neuronal cells.

### BRD4 inhibition improved cognitive impairment in ethanol-fed mice

**Figure 7:**
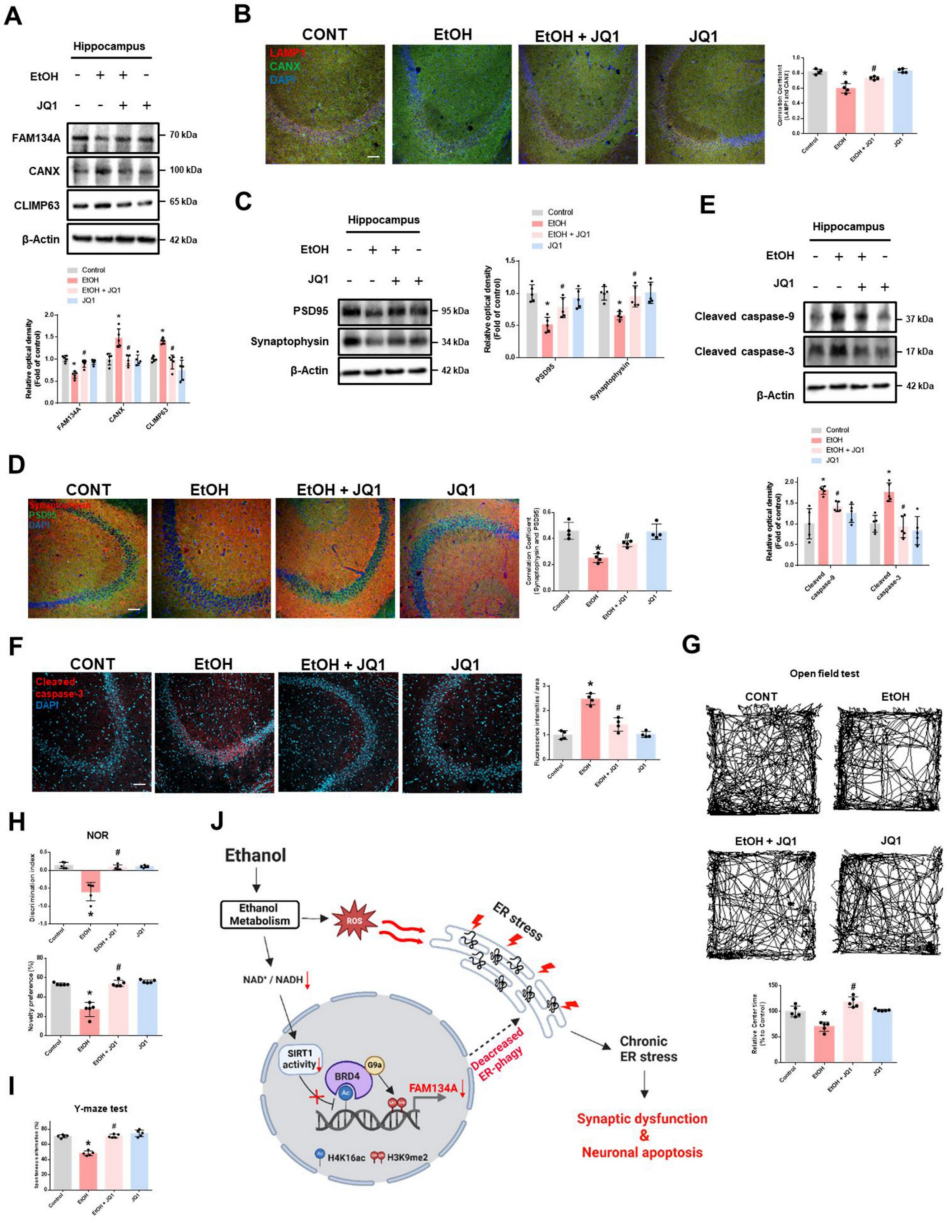
** Inhibition of BRD4 prevents synaptic dysfunction, neuronal apoptosis, and cognitive impairment in ethanol-fed mice. (A-I)** The experimental mice were treated with vehicle, ethanol, ethanol with JQ1, and JQ1 alone for 4 weeks. Samples were obtained from mice hippocampus. **(A)** Hippocampal FAM134A, CANX, and CLIMP63 were detected by western blot. *n* = 5. **(B)** Immunohistochemistry images showing LAMP1 and CANX. Scale bars, 20 μm. *n* = 4.** (C)** Hippocampal PSD95 and Synaptophysin were detected by western blot. *n* = 5.** (D)** Hippocampal IHC slides were immunostained with PSD95 and Synaptophysin. Scale bars, 20 μm. *n* = 4.** (E)** Protein levels of the hippocampal cleaved caspase-9 and cleaved caspase-3 were investigated. *n* = 5. **(F)** Tissue slides for immunohistochemistry were immunostained with cleaved caspase-3 -specific antibody. Scale bars, 20 μm. *n* = 4. **(G-I)** The mice were subjected to open field test, NOR test, and Y-maze test, respectively. n = 5. **(J)** The schematic model for inhibition of BRD4-mediated FAM134A-dependent ER-phagy by ethanol was shown. All data are representative. Quantitative data are presented as a mean ± SD. * indicates *p* < 0.05 versus control, # indicates *p* < 0.05 versus EtOH.

We evaluated the neuroprotective effects of BRD4 inhibition in the hippocampus of ethanol-fed mice. Ethanol reduced FAM134A protein levels and ER-phagy, but these effects were prevented by JQ1 treatment in the hippocampus (Fig. 7A-B). In addition, JQ1 injection mitigated the inhibitory effects of ethanol on hippocampal synaptic function and cell viability (Fig. 7C-F). We then assessed whether pharmacological BRD4 inhibition improves ethanol-induced memory impairment using the open field test, novel object recognition (NOR), and Y-maze in ethanol-fed mice. In the open field test, ethanol-fed mice spent less time in the center of the open field compared to other mice groups (Fig. 7G). Notably, we observed that ethanol-fed mice with JQ1 treatment recovered cognitive function compared with ethanol-fed mice (Fig. 7H-I). Collectively, these findings suggest that pharmacological inhibition of BRD4 may alleviate ethanol-induced synaptic deficits, neuronal apoptosis, and cognitive impairment.

## Discussion

This study identifies ER-phagy dysfunction as a potential pathophysiological feature of ethanol-related neurological disorders. Our *in vitro* experiments demonstrated that ethanol impairs FAM134A-mediated ER-phagy, thereby promoting neurodegeneration. *In vivo* findings further confirmed that synaptic loss and neuronal death, both of which contribute to cognitive impairment, were alleviated by restoring FAM134A expression through BRD4 inhibition in ethanol-fed mice. Ethanol-induced ROS production leads to the accumulation of unfolded or misfolded proteins in the ER, resulting in ER stress 36. Previous studies have established that ER stress plays a central role in the pathogenesis of neurodegenerative diseases and that its inhibition reduces ethanol-induced apoptotic neurodegeneration in both developing and adult brains 37-39. Recently, dysfunction of ER-phagy receptors has been linked to the development of neurodegenerative disorders 40, 41. Mutations in *FAM134B* and *ATL3* are associated with hereditary sensory and autonomic neuropathy types II and I, respectively 42, 43. *RTN3* deficiency has been implicated in Alzheimer's disease (AD) 44, and decreased *TEX264* expression has been observed in AD-associated neuroinflammation 45. These findings indicate that impaired function of ER-phagy receptors may contribute to chronic ER stress and subsequent neurodegeneration under ethanol exposure. In the present study, we demonstrated for the first time that ethanol significantly downregulates *FAM134A* mRNA and protein expression among ER-phagy receptors. Since FAM134A is localized to the ER sheet, where protein synthesis and folding occur, downregulated FAM134A-mediated ER-phagy may not sufficiently relieve the ethanol-induced accumulation of unfolded proteins, thereby contributing to chronic ER stress in neuronal cells.

We investigated the regulatory mechanism of *FAM134A* gene expression in neuronal cells exposed to ethanol. Although previous studies have shown that inhibition of BET protein enhances LC3 lipidation and autophagosome formation 46-48, it remains unclear whether BRD4 specifically regulates selective forms of autophagy. Given previous reports that ethanol metabolism directly contributes to histone acetylation 20, 49, along with our observation that ethanol suppresses ER-phagy, we hypothesized that BRD4 may regulate the expression of ER-phagy-related genes in ethanol-exposed neuronal cells. Our findings showed that ethanol increases both BRD4 occupancy and H4K16ac at the *FAM134A* promoter. Notably, pretreatment with a SIRT1 activator reduced BRD4 binding to the *FAM134A* promoter, which had been enhanced by ethanol. These results indicate that BRD4 may repress *FAM134A* transcription by binding to ethanol-induced H4K16ac at its promoter. H4K16ac, a major histone substrate of SIRT1, has been reported to be important in regulating the expression of autophagy-related genes 31, 50. Although the exact mechanism has not been elucidated, the role of H4K16ac in regulating autophagy-related genes is controversial. Some studies have reported that a decrease in H4K16ac levels leads to downregulation of ATG genes 51, 52. Another report revealed that increases in H4K16ac levels were associated with transcriptional repression of the LC3B gene 53. Furthermore, a study found that reduced H4K16ac promotes autophagy by increasing ATG gene transcription in retinal cell lines 54. These discrepancies indicate that the effect of H4K16ac on autophagy gene regulation may be context-dependent, influenced by the specific cell type, experimental conditions, and interacting cofactors. Therefore, our findings suggest that ethanol metabolism contributes to increased H4K16ac levels on the *FAM134A* promoter, and BRD4, which recognizes and binds to it, may repress *FAM134A* transcription by forming complexes with other regulatory proteins in neuronal cells.

This study demonstrated that ethanol increases G9a protein expression and promotes the formation of a BRD4-G9a complex. Furthermore, the ethanol-induced increase in H3K9me2 levels on the *FAM134A* promoter was reduced by inhibiting either BRD4 or G9a. These findings suggest that BRD4 and G9a cooperatively repress *FAM134A* expression via H3K9me2-mediated chromatin modification. Consistent with our results, several studies have shown that BRD4 interacts with histone-modifying enzymes to regulate gene expression. For instance, BRD4 has been reported to interact with LSD1 to demethylate H3K4me1/2, thereby repressing genes such as *GNA13* and *PDPK1* 33. Another study found that BRD4 interacts with the enhancer of EZH2 to inhibit the transcriptional expression of Foxp3 and Fbxw7 by promoting H3K27me3 32. However, under our experimental conditions, we observed no significant changes in EZH2 or LSD1 expression. Although interactions between BRD4 and other histone modifying enzymes cannot be completely ruled out, our data suggest that G9a may play an important role in BRD4-mediated gene repression. Additionally, given the co-occurrence of increased H3K9me2 and H4K16ac at the same FAM134A promoter region, we propose that these two histones' modifications may coexist and act cooperatively to repress gene expression. BRD4 may serve as a central mediator, recognizing H4K16ac while recruiting G9a to deposit H3K9me2, thereby establishing a transcriptionally repressive chromatin environment. Although BRD4-mediated histone modifications play a pivotal role in repressing *FAM134A* expression, transcription factors activated by UPR signaling may also contribute to its regulation. Bernales et al. reported that ER-phagy is triggered during ER remodeling via UPR signaling to alleviate stress caused by accumulation of unfolded proteins 55. Moreover, ATF4 has been shown to induce *FAM134B* and *TEX264* expression during loperamide-induced ER stress 9, supporting a broader role for UPR-related transcription factors in ER-phagy regulation. In our analysis of the *FAM134A* promoter, we identified multiple XBP1s binding motifs, more than those for other UPR-associated transcription factors, suggesting that XBP1s exert a stronger regulatory influence on *FAM134A* expression. Importantly, we found that ethanol reduced XBP1s binding to the *FAM134A* promoter, and this repression was reversed by BRD4 inhibition. These results indicate that despite the activation of XBP1s via ethanol-induced UPR signaling, increased H3K9me2 may restrict its promoter access through chromatin condensation. Together, we propose that the BRD4-G9a complex plays a central role in regulating *FAM134A* expression in ethanol-exposed neuronal cells.

Although no prior studies have reported a biological role for FAM134A in nervous system cells or tissues, our findings are the first to demonstrate that restoring FAM134A-mediated ER-phagy suppresses ethanol-induced ER stress and mitigates neurodegeneration. Interestingly, a recent study reported that FAM134A, unlike other ER-phagy receptors, mediates ER-phagy through interactions with both LC3 and GABARAP 56. LC3 generally participates in early autophagosome formation, while GABARAP plays a crucial role in later steps, such as autophagosome-lysosome fusion 57. These findings suggest that FAM134A may function in both the initiation and maturation phases of ER-phagy. Based on previous and present findings, we propose that FAM134A alleviates ethanol-induced ER stress by participating in both the autophagosome and autolysosome formation processes of the ER sheet. Furthermore, our study is the first to demonstrate that DDRGK1-mediated UFMylation may regulate FAM134A-dependent ER-phagy activation. These findings highlight that not only the expression level of the ER-phagy receptors but also their post-translational modifications (PTMs), are critical for ER-phagy initiation. Recent studies have suggested that other PTMs, such as phosphorylation and ubiquitination, also modulate the activity of ER-phagy receptors 35, 58, 59. Therefore, further research is needed to determine whether UFMylation influences oligomerization of FAM134A or whether other PTMs are involved in its activation.

A previous study reported that BRD4 inhibition affects essential synaptic proteins, leading to memory impairment and simultaneously reducing seizure susceptibility in mice 60. Conversely, other studies have shown beneficial effects of BRD4 inhibition. For example, JQ1 has been found to enhance cognitive performance and long-term potentiation in AD animal model 61. Furthermore, a study reported that JQ1 treatment suppressed hippocampal apoptosis and improved cognitive impairment in diabetic rats 62. In our study, JQ1 restored hippocampal FAM134A protein levels, which enhanced ER-phagy and, in turn, inhibited ethanol-induced neurodegeneration. Although the protective effect of BRD4 inhibition on cognitive impairment is controversial according to experimental conditions, our findings suggest that JQ1 administration mitigates synaptic dysfunction, neuronal cell death, and cognitive impairment by restoring FAM134A-mediated ER-phagy in ethanol-fed mice. Here, our findings indicate that BRD4 is a potential target for alleviating ethanol-induced neuronal damage caused by sustained ER stress. These results contribute to understanding of the intricate interplay between ethanol and ER-phagy and may aid in developing prevention strategies and targeted treatments for ethanol-related diseases. In conclusion, our study highlighted that ER-phagy reduced by BRD4-mediated FAM134A downregulation in neurons exposed to ethanol induces chronic ER stress, resulting in synaptic dysfunction, neuronal apoptosis and cognitive impairment (Fig. 7J).

## Supplementary Material

Supplementary figures.
