# Supplementary material for: Recovery of FAM134A-mediated ER-phagy through BRD4 inhibition alleviates ethanol-induced neurodegeneration
Source: Int J Biol Sci. 2025 Aug 11;21(12):5167–84. doi: 10.7150/ijbs.116673 (PMC12435209; doi:10.7150/ijbs.116673)
Supplement: Supplementary file 1 — Supplementary figures. [file ijbsv21p5167s1.pdf]

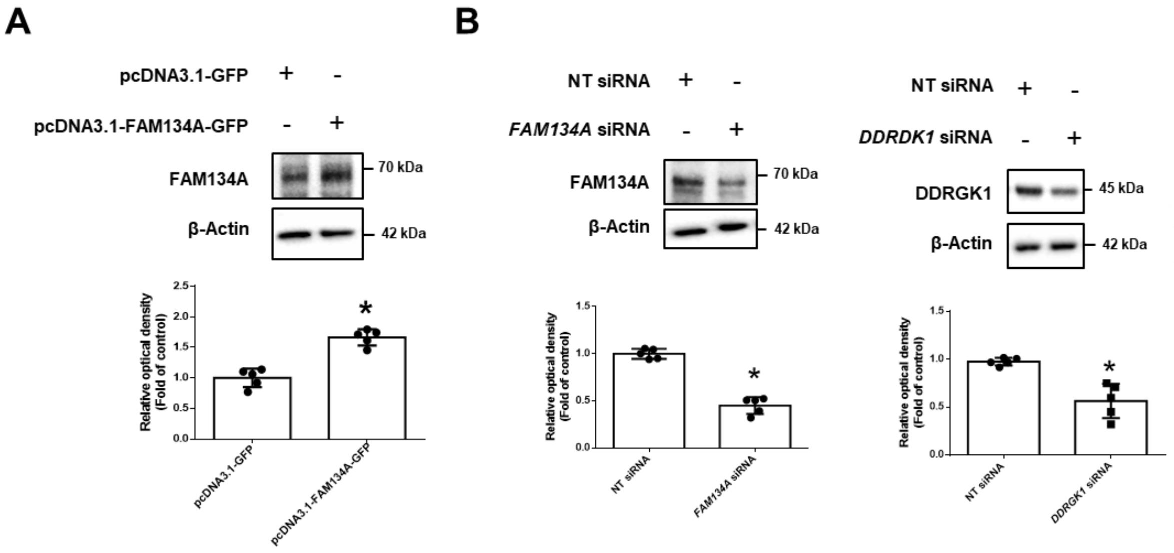

Supplementary Fig 1. Confirmation of the efficiency of siRNA and plasmid DNA.

(A) SH-SY5Y cells were transfected with pcDNA3.1-FAM134A-GFP for 24 h, $n = 5$. (B) Cells were transfected with either FAM134A siRNA or DDRGK1 siRNA for 24 h, respectively. $n = 5$. All blot images are representative. Quantitative data are presented as a mean ± SD. * indicates $p < 0.05$ versus control.

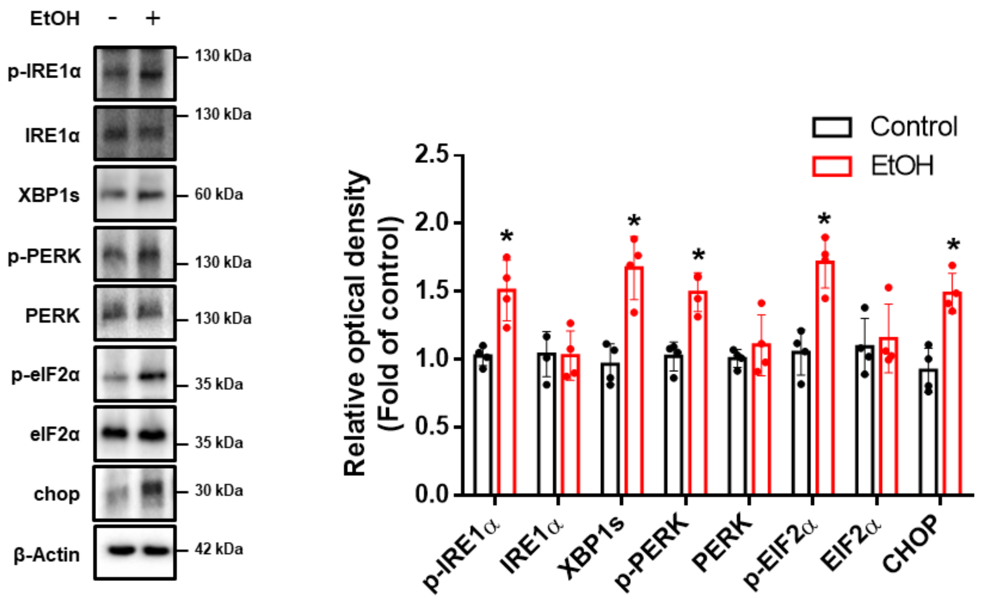

Supplementary Fig 2. Effect of ethanol on ER stress.

EtOH was treated to SH-SY5Y cells for 24 h. The levels of ER stress marker proteins were measured by western blotting. $n = 4$. All blots are representative. Quantitative data are presented as a mean ± SD. * indicates $p < 0.05$ versus control.

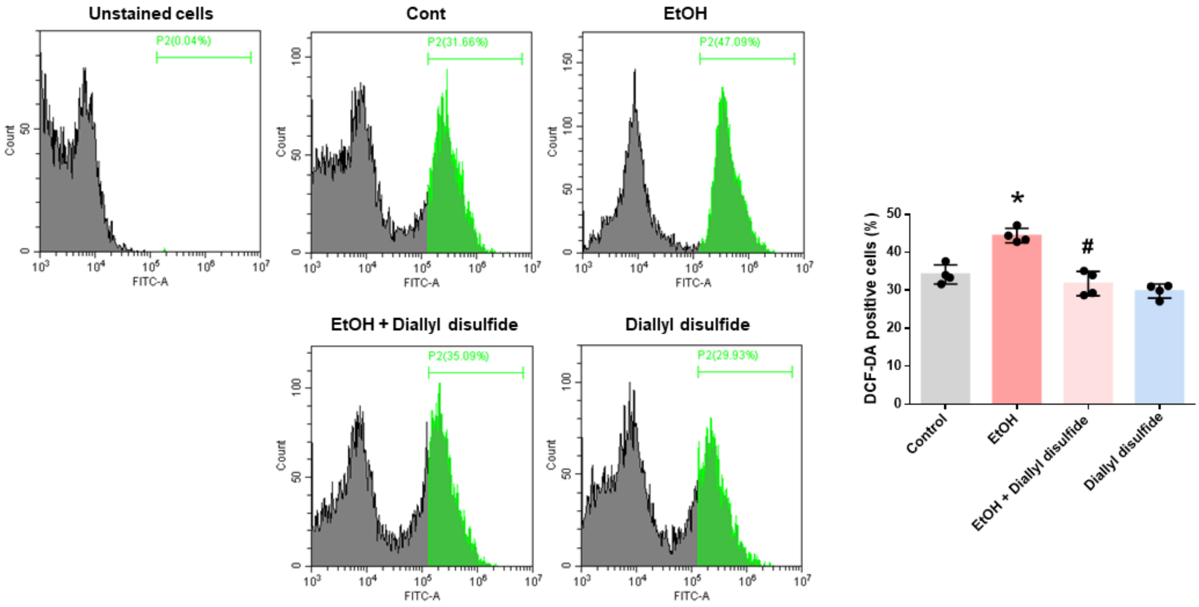

Supplementary Fig 3. Effect of ethanol metabolism on ethanol-induced ROS production.

Diallyl disulfide (10 μM) was applied for 30 min prior to the treatment of EtOH for 24 h in SH-SY5Y cells. H_2DCF-DA staining was performed to detect ROS and it was analyzed with flow cytometer. $n = 4$. Flow cytometer images are representative. Quantitative data are presented as a mean ± SD. * indicates $p < 0.05$ versus control, # indicates $p < 0.05$ versus EtOH.

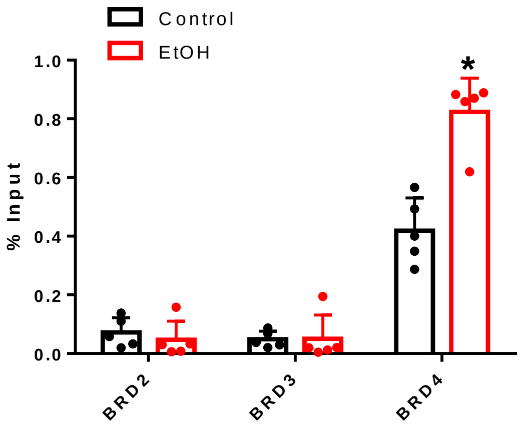

Supplementary Fig 4. Effect of ethanol on BRD proteins binding to the FAM134A promoter.

iPSC-neurons were exposed to EtOH for 12 h. DNA was immunoprecipitated with antibodies against BRD2, BRD3, and BRD4, respectively. The immunoprecipitation samples were amplified with primer of *FAM134A* gene. Data were obtained using qPCR. $n = 5$. Quantitative data are presented as a mean ± SD. * indicates $p < 0.05$ versus control.

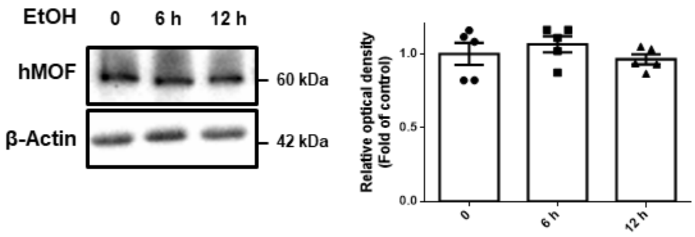

Supplementary Fig 5. Effect of ethanol on hMOF expression.

EtOH was treated for 0, 6, and 12 h to iPSC-neurons. Western blotting was conducted to determine the levels of hMOF. $n = 5$. Blot images are representative. Quantitative data are presented as a mean ± SD.

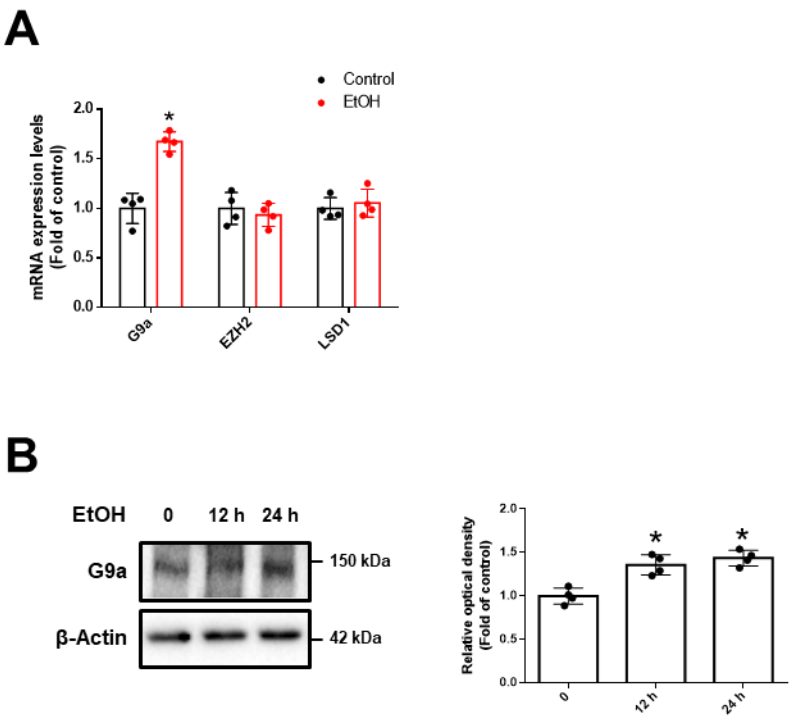

Supplementary Fig 6. Effect of ethanol on the expression of histone-modifying enzymes.

(A) EtOH was treated to iPSC-neurons for 12 h. The mRNA expression levels of G9a, *EZH2, and LSD1* were analyzed by real-time PCR. $n = 4$. (B) iPSC-neurons were treated with EtOH for 0, 12, and 24 h. Protein expression level of G9a was analyzed by western blotting. $n = 4$. Blot images are representative. Quantitative data are presented as a mean ± SD. * indicates $p < 0.05$ versus control.

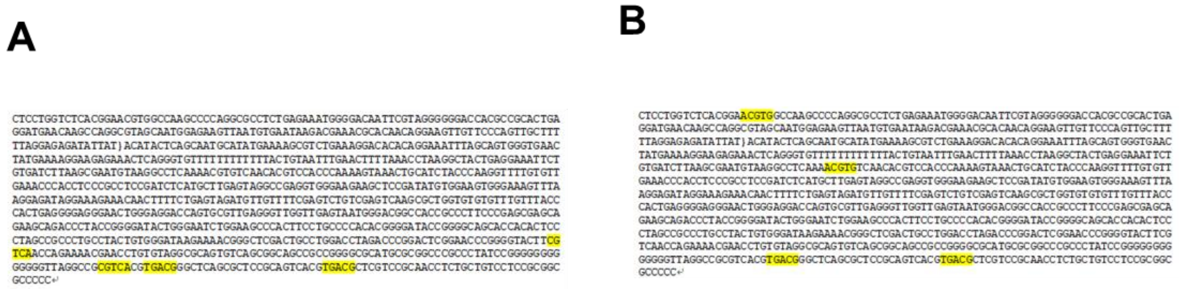

Supplementary Fig 7. Binding sequences of ATF4 and ATF6 in FAM134A promoter regions.

(A-B) The thousand base pair upstream of the first codon of FAM134A was described and the putative ATF4 and ATF6 binding sequences were emphasized with yellow labeling, respectively.

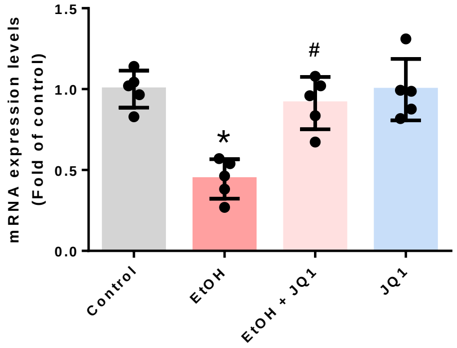

Supplementary Fig 8. Effect of JQ1 on ethanol-inhibited FAM134A mRNA expression.

JQ1 (25 nM) was applied for 30 min prior to the treatment of EtOH for 12 h in iPSC-neurons. The mRNA expression levels of *FAM134A* were analyzed by real-time PCR, $n = 5$. Quantitative data are presented as a mean ± SD. * indicates $p < 0.05$ versus control, # indicates $p < 0.05$ versus EtOH.

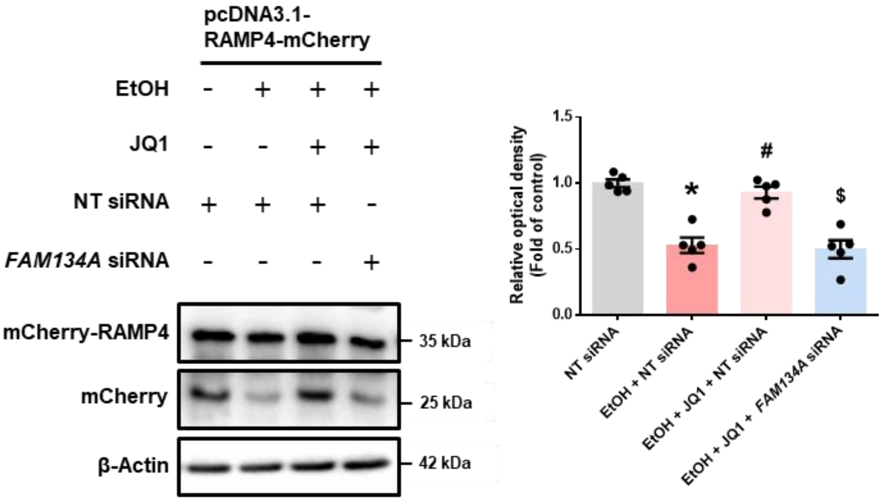

Supplementary Fig 9. Role of FAM134A in restoring ER-phagy through BRD4 inhibition.

Cells were transfected with pcDNA3.1-RAMP4-mCherry and either NT siRNA or FAM134A siRNA, then pretreated with JQ1 (25 nM) for 30 min prior to treatment with EtOH for 24 h. The protein expression of mCherry-RAMP4 and mCherry were detected by western blotting. $n = 5$. Blot images are representative. Quantitative data are presented as a mean ± SD. * indicates $p < 0.05$ versus NT siRNA, # indicates $p < 0.05$ versus EtOH + NT siRNA, $ indicates $p < 0.05$ versus EtOH + JQ1 + NT siRNA.

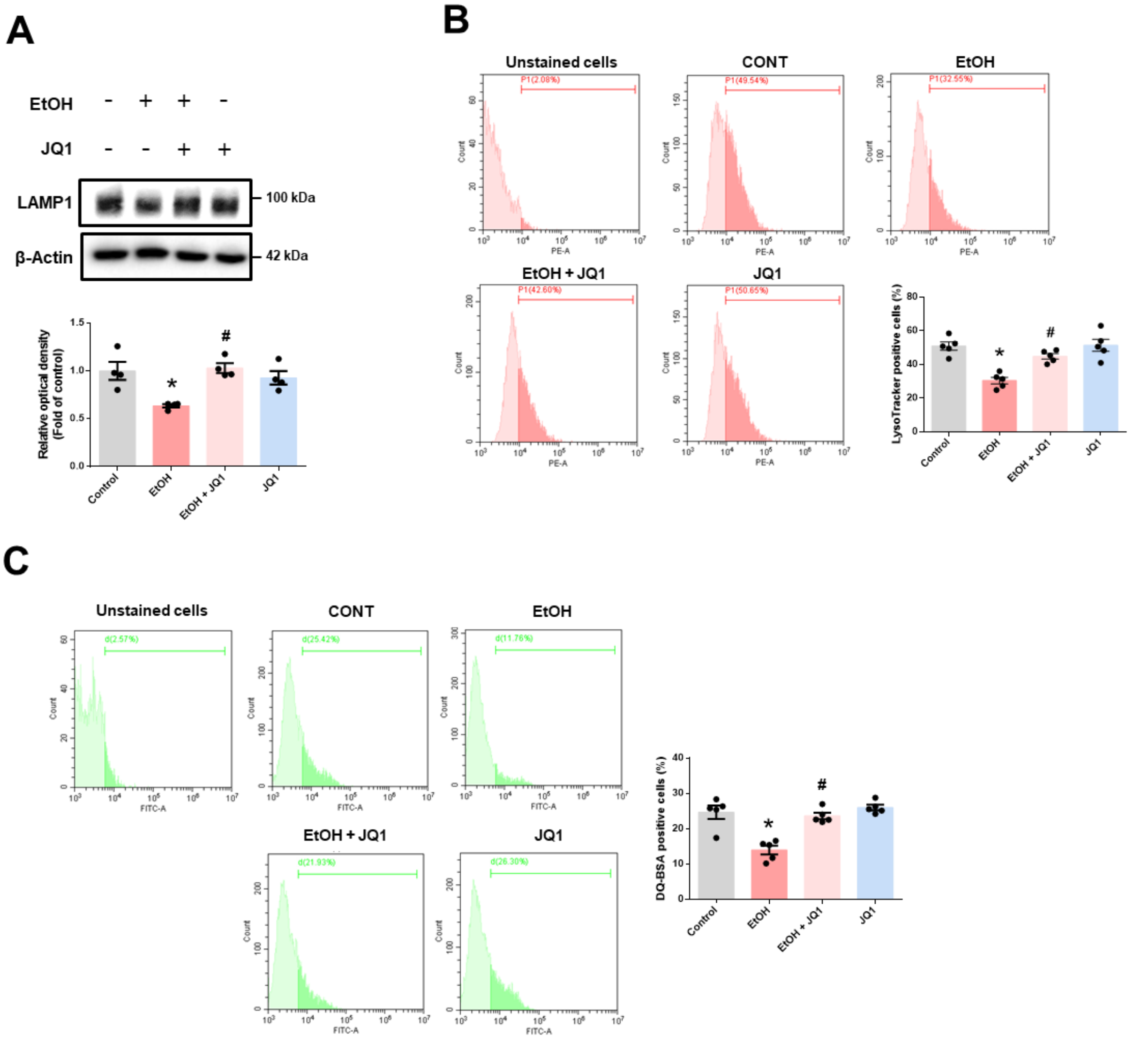

Supplementary Fig 10. Effect of JQ1 on ethanol-inhibited lysosome biogenesis.

(A-C) JQ1 (25 nM) was applied for 30 min prior to the treatment of EtOH for 24 h in iPSC-neurons. (A) LAMP1 was detected by western blotting. $n = 4$. (B-C) LysoTracker- and DQ-BSA-positive cells were analyzed with flow cytometer. $n = 5$. All data are representative. Quantitative data are presented as a mean ± SD. * indicates $p < 0.05$ versus control, # indicates $p < 0.05$ versus EtOH.

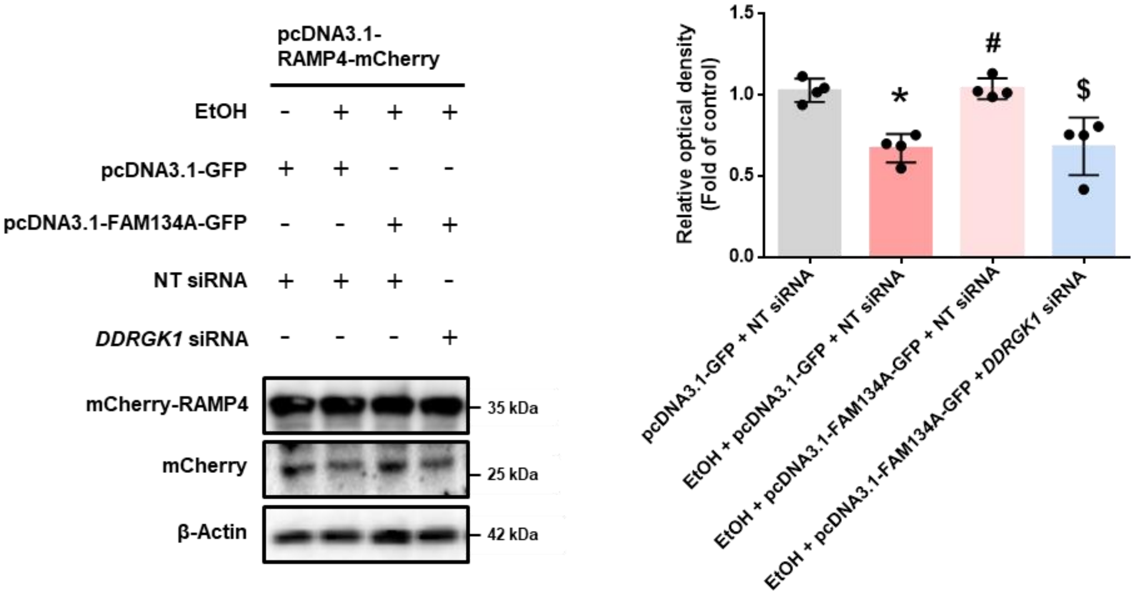

Supplementary Fig 11. DDRGK1-mediated UFMylation regulates FAM134A-mediated ER-phagy in neuronal cells exposed to ethanol.

After CCER system construction, cells were transfected with pcDNA3.1-GFP or pcDNA3.1-FAM134A-GFP and NT siRNA or DDRGK1 siRNA. Cells were incubated with EtOH for 24 h. mCherry-RAMP4 and mCherry protein expressions were detected by western blotting. $n = 4$. Blot images are representative. Quantitative data are presented as a mean ± SD. * indicates $p < 0.05$ versus pcDNA3.1-GFP + NT siRNA, # indicates $p < 0.05$ versus EtOH + pcDNA3.1-GFP + NT siRNA, $ indicates $p < 0.05$ versus EtOH + pcDNA3.1-FAM134A-GFP + NT siRNA.
